# A Guided Web-Based Intervention Targeting Procrastination in College Students: Protocol for an Open Trial

**DOI:** 10.2196/44907

**Published:** 2023-11-03

**Authors:** Sevin Ozmen, Arpana Amarnath, Sascha Struijs, Leonore de Wit, Pim Cuijpers

**Affiliations:** 1 Department of Clinical Psychology Vrije Universiteit Amsterdam Amsterdam Netherlands

**Keywords:** eHealth, feasibility, guidance, online intervention, procrastination, protocol

## Abstract

**Background:**

Academic procrastination is a widespread problem among college students. It is linked to poor academic performance and increased college dropout intentions, as well as several mental health problems such as depression, anxiety, and stress. Guided web-based interventions can help reduce procrastination. However, guidance by professional clinicians draws upon valuable and limited societal resources, and a more efficient, scalable form of guidance is needed. Guidance by trained clinical psychology students has not yet been examined.

**Objective:**

The aim of this open trial is to examine the feasibility and acceptability of a web-based procrastination intervention for college students under the guidance of student digital coaches (e-coaches).

**Methods:**

We developed a single-arm trial of a guided web-based intervention targeting procrastination for the Dutch student population. Guidance is delivered by trained clinical psychology students asynchronously in the form of textual feedback on intervention progress, with the aim of supporting and motivating the participant. Participants are recruited at 7 Dutch universities. Primary outcomes are intervention satisfaction, usability, and adherence, which are assessed by the Client Satisfaction Scale (CSQ-8), System Usability Scale (SUS-10), and number of completed modules, respectively. The primary outcomes will be examined by calculating descriptive statistics. Secondary outcomes are e-coach satisfaction and changes to procrastination, depression, stress, and quality of life from pre- to posttest and follow-up.

**Results:**

The project was funded in 2019, and recruitment began in January 2021. As of May 2023, a total of 985 participants were enrolled, of which 372 had completed the posttest and 192 had completed the follow-up. The expected date of analysis and publication of the results is 2024.

**Conclusions:**

The results are expected to contribute to the body of literature regarding eHealth in 3 ways. First, we will examine whether students who procrastinate adhere to and are satisfied with an eHealth intervention targeting this problem. Second, we will explore whether an intervention targeting procrastination can also decrease depression and stress. Lastly, we will investigate whether trained psychology students can effectively guide their peers in web-based interventions. Given the shortage of licensed psychologists, exploring alternative sources of guidance is much needed in order to provide students with the mental health support they need.

**International Registered Report Identifier (IRRID):**

DERR1-10.2196/44907

## Introduction

Academic procrastination is a widespread problem among college students, with studies reporting prevalence rates ranging from 29% to 67% [1-4]. Procrastination, which is defined as the tendency “to voluntarily delay an intended course of action despite expecting to be worse off for the delay” [5], is linked to poor academic performance [6] and increased college dropout intentions [7]. Procrastination is also associated with several mental health problems, such as depression [8], anxiety, stress [9,10], maladaptive coping, and low self-esteem [11]. Negative long-term consequences associated with procrastination include poorer overall health, difficulties with social relationships, financial struggles, and limited career opportunities [10,12,13].

Effective treatments to target procrastination are available. A recent meta-analysis reports that psychological treatments are effective in reducing procrastination [14]. When comparing different types of treatment, cognitive behavioral therapy (CBT) was found to be the most effective in reducing procrastination when compared with self-regulation training, strengths training, and acceptance-based behavior therapy [15].

Still, it is uncommon for students to seek or receive help for mental health complaints due to several barriers. The most common barriers are the perception that help is not needed, as students view their problems as minor or temporary, and a perceived lack of time. Other barriers include a preference for self-management, a fear of stigmatization, and low awareness of available resources [16,17]. Lastly, the detection and treatment of students with mental issues in general is hampered by limited available resources and a limited number of available student psychologists or student counselors, resulting in long waiting lists [18]. One solution to several of these barriers may be eHealth interventions, consisting of web-based treatments such as CBT. They offer quick access to treatment, anonymity, flexibility, and improved cost-effectiveness compared to face-to-face treatment [19,20]. These interventions are effective in improving mental well-being for a wide range of psychological complaints such as depression, anxiety [21,22], and stress [23]. Guided eHealth and face-to-face treatment have been found to be equally effective in treating depression and anxiety [24,25].

Studies examining the effectiveness of eHealth interventions targeting procrastination specifically show that eHealth is also effective in reducing procrastination [26-28]. When comparing eHealth to traditional treatment, chat-based counseling and face-to-face counseling resulted in a comparable decrease in procrastination [29]. Similarly, a group intervention and self-guided internet-based treatment produced comparable levels of improvement for procrastination behavior after the treatment period [30].

However, there are also drawbacks to eHealth. An often-mentioned reason for a lessened intervention effect is the problem of nonadherence, meaning participants do not make optimal use of the intervention [31-33]. For procrastination specifically, the problem of nonadherence could be exacerbated, as individuals with procrastination tendencies can struggle to adhere to the treatment [28].

Adding therapist guidance to eHealth interventions could offer a solution to this problem. Meta-analytic evidence suggests that guidance from professionals increases eHealth treatment effectiveness when compared with unguided treatment [24,34]. This may be explained by the positive effect guidance has on motivation, engagement, and adherence [35-37]. However, the addition of professional guidance in eHealth draws upon valuable and limited societal resources, and there is a scarcity of properly trained professionals.

The question arises whether there is a more efficient, scalable form of guidance without lowering therapy effectiveness. There is evidence suggesting guidance by nonclinicians (eg, peers, research assistants, or other laypeople) has comparable effects compared to guidance by clinicians in eHealth interventions [38]. What has not specifically been examined yet is whether guidance by trained clinical psychology university students can be equally effective. Were this to be the case, it could greatly reduce the load on the health care system as trained clinical psychology students could offer eHealth guidance for significantly lower costs compared to clinicians. Finally, most clinical psychology students welcome the opportunity to gain hands-on experience guiding real clients during their studies.

The main objective of this study is to evaluate the acceptability and feasibility of a new eHealth intervention targeting procrastination in college students that is guided by clinical psychology students by assessing its usability, adherence, and client satisfaction. The secondary objectives are to explore differences between pre- and posttests for procrastination, depression, stress, and quality of life.

## Methods

### Study Design

This study is a single-arm open trial of a guided eHealth intervention (GetStarted) aimed at decreasing procrastination in college students. It uses a single-group pretest (T0), posttest (T1), and follow-up (T2) design in which T1 and T2 are administered 4 weeks and 6 months after T0, respectively. The study is conducted within the Caring Universities consortium, a Dutch project that is part of the World Mental Health International College Student Initiative (WMH-ICS) of the World Health Organization (WHO) [39].

### Participants and Recruitment

Participants are undergraduate, graduate, or PhD students enrolled in the abovementioned 7 universities across the Netherlands. These 7 universities are situated in 10 cities across the Netherlands and have a combined total of approximately 190,000 domestic and international students. Participants may be both domestic and international students.

The aim of this study is to recruit a minimum of 50 students, and recruitment is conducted in 3 ways. Firs, through a yearly web-based survey of the WMH-ICS. This survey canvasses the mental well-being of students and is sent by email to all the students of the participating universities. At the end of the survey, students who want to receive feedback are provided feedback on their mental health. Students who show increased procrastination tendencies (ie, a score >28 on the Irrational Procrastination Scale; IPS) are invited to sign up for the Caring Universities procrastination program. Second, marketing activities are conducted on the web through social media and on campus through posters, flyers, and other advertisements. Third, we recruit participants through the staff at participating universities. Staff members who work directly with students, such as student psychologists, study advisors, and lecturers, are informed of the program and can recommend it to students who might be interested.

Students are not offered compensation for participation.

### Eligibility Criteria

Participants will be included if they meet the following criteria: (1) being aged 16 years or older; (2) being enrolled as a bachelor, master’s, or PhD student; and (3) having given informed consent.

The sole exclusion criterion is suicidal risk. Participants will be asked if they have had thoughts of killing themselves or made plans to kill themselves in the past 12 months. If they answer yes, the follow-up question, “How likely do you think it is that you will act on this plan in the next 12 months?” is asked. Participants that respond to this question with “somewhat likely” or “very likely” are excluded from the intervention.

### Intervention

The guided eHealth program aimed at reducing procrastination behavior (GetStarted) is developed based on the principles of CBT. Students are involved in the development of the program by giving their opinions and feedback on the program content as well as the layout of the website that contains the program. The feedback is implemented to ensure that GetStarted meets students’ needs and preferences.

**Figure 1:**
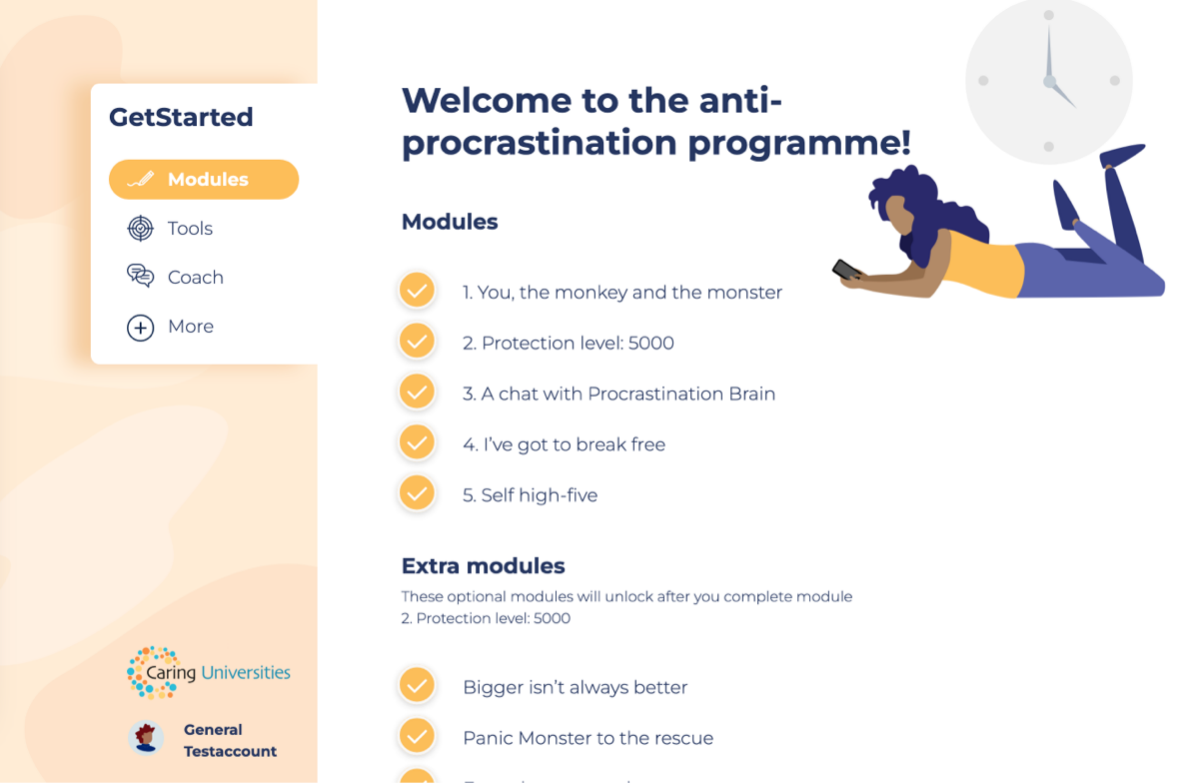
Screenshot of GetStarted, an eHealth intervention targeting procrastination.

GetStarted (Figure 1) consists of 9 modules, of which 5 are mandatory main modules and 4 are optional modules. The modules consist of psychoeducation, reflective questions, interactive exercises, and homework assignments. The content is delivered both textually and visually, including through the use of pictures, infographics, and videos. Each module takes approximately 30 to 45 minutes to complete, and students are advised to complete 1 main module a week. The duration of the program will therefore be approximately 4-5 weeks, though students are allowed to go at their own pace and can access the intervention for 2 years. Students will be able to access the program from any device that allows internet access.

The first module offers an introduction to the program and its contents. Students are recommended to continue with the second module right away, which covers information on the psychological processes behind procrastination. The third module will allow students to identify the negative thoughts and feelings that accompany certain tasks, causing them to procrastinate. The fourth module will contain a cognitive restructuring exercise in which they will challenge their negative thinking patterns. The fifth and final main module will let the student reflect on their progress, prepare for the future, and include a summary of all the modules.

In addition to the main modules, students will be able to choose to do 1 or several of 4 optional modules. The topics of the optional modules will be (1) breaking a big task into smaller parts; (2) creating and sticking to a plan; (3) motivational techniques; and (4) increased productivity. All optional modules will become available upon completion of the second main module.

The intervention will be created based on the following principles of optimized user interface [40] in collaboration with a user experience designer: a menu containing several main sections, presentation of content in small chunks, stepwise exercises with separate instructions at each step, examples presented in expandable containers, and showing responses given by the participant in previous exercises when relevant.

### Digital Coaches

During the registration process, participants select a digital coach (ie, e-coach) to support them during the program. The e-coach provides asynchronous, textual feedback through the platform after the completion of every module by the participant. While doing the intervention, participants respond to questions about the subject of procrastination. The e-coaches read these answers, which form the basis of the textual feedback. In this feedback, e-coaches respond to the participant’s answers, show empathy, encourage the participant to apply the techniques, and ask reflective questions with the aim of deepening the participant’s understanding of their procrastination. The feedback contains the following elements:

- an opening statement containing positive feedback for the participant’s completion of the module;
- selecting 2 to 3 points of note in the participant’s answers to provide empathy, encouragement, reiterate the goal of the module, or ask a follow-up question; and
- a closing statement regarding the next module.

Writing feedback for a single module takes around 30 minutes on average, and e-coaches provide feedback within 3 working days of a module being completed by the participant. E-coaches also send reminders about the intervention to participants who have been inactive for a while. E-coaches who cannot provide feedback within 3 working days due to a short temporary absence (such as a holiday) write an explanatory message to the participants explaining the absence and informing them of a return date. In the event that an e-coach cannot adequately perform their duties in the long term, the participant is assigned a different available e-coach. The reason for the change, as well as an apology, are provided to the participant by the new e-coach. A member of the research team monitors the e-coaches and the quality of the coaching.

E-coaches are (research) master students in clinical psychology and third-year clinical psychology bachelor students who meet a number of requirements in the area of low-intensity treatments for common mental health problems. E-coaches are recruited by making third-year bachelor and master students aware of the opportunity to coach as an (extracurricular) internship. They can apply for the position with their curriculum vitae and motivation. Based on their previous experience and motivation, certain students are then invited for an interview with the research team, and selected students are offered the position. These students complete 6 hours of coach training before starting their coaching activities and are required to attend weekly 1-hour intervision meetings, which are supervised by the research team.

### Possible Harms

Several meta-analyses show that internet-based programs might be beneficial for college students [41] and that there are no indicated risks to internet-based programs. Moreover, individual patient data meta-analyses showed that participants who received an internet-based program for depression had a lower risk of clinical deterioration when compared with the control group [20,42]. These findings seem to indicate minimal to no risk of possible harm. However, in the event that a participant shows signs of more severe mental health problems, which include but are not limited to self-harm or suicidality, in their responses to the questions in the intervention, the protocol for (emerging) crisis situations will be applied: the e-coach (under the supervision of and in collaboration with a certified mental health care psychologist) will reach out and assess the student’s current situation in more detail through the platform. In cases of mild to moderate risk of a worsening of psychological symptoms, we will advise the student to seek professional help aside from participating in the study. In cases of high risk, we advise the student to seek out professional help and resolve the current situation before continuing participation in the study.

### Platform

The intervention is embedded within the Caring Universities platform. This platform was developed by Caring Universities specifically to deliver eHealth interventions to college students. The platform allows researchers to create interventions, add and arrange the contents (eg, text and visuals), and add questionnaires. Students of participating universities can freely register for the intervention by creating an account on their device (eg, computer or mobile phone). Participants can log into the platform to view its contents 24-7 for a total duration of 2 years, even upon completion of the program. During registration, each participant is presented with a random selection of 3 e-coaches out of all available coaches to choose from based on a name, short biography, and profile picture. Within the platform, participants and e-coaches can interact using textual messages. The platform complies with the General Data Protection Regulation (GDPR) guidelines.

### Assessment Measures

#### Primary Outcomes

##### Satisfaction With the Intervention

To measure participants’ satisfaction with the overall intervention, the Client Satisfaction Questionnaire–8 (CSQ-8) [43] is used. The CSQ-8 is commonly used to measure satisfaction with web-based interventions. It consists of 8 items on a 4-point scale with a total score ranging from 8 to 32, where a higher score indicates greater satisfaction. The CSQ-8 showed high reliability and validity for web-based interventions [44]. Similar properties were found in the Dutch translation of the CSQ-8 [45].

##### Usability

The System Usability Scale–10 (SUS-10) [46] is used to measure the usability of the intervention. It consists of 10 items on a 5-point Likert scale, with a total score ranging from 0 to 40. Total scores are then multiplied by 2.5 to achieve a total score of 0 to 100, where a higher score indicates greater usability. The SUS-10 has become the most widely used standardized questionnaire to assess perceived usability and has shown good psychometric properties (reliability and validity) [47]. The Dutch translation of the SUS-10 has previously been used [48], though publications on its specific psychometric properties could not be found. However, several other translations of the SUS-10 were found to have similar psychometric properties as the English version [47].

##### Adherence

Adherence refers to “the degree to which the user followed the program as it was designed” [32]. This study measures adherence by dividing the number of main modules completed by a participant at the time of posttest by the total number of main modules in the program and multiplying this by 100. The resulting percentage will indicate the completion rate. Participants who have not completed a single module will be excluded from these analyses as they did not start the treatment.

#### Secondary Outcomes

##### Procrastination Tendencies

The IPS is used to assess procrastination tendencies [49,50]. This scale contains 9 items on a 5-point Likert scale. The scale aims to assess the extent to which participants procrastinate. Higher scores indicate higher levels of procrastination. The IPS is used to assess the changes from pre- to postscores. The IPS showed good internal consistency (Cronbach α=.91) [50] as well as a high level of reliability (*r_pm_*=0.58-0.74), and good content, structural, and substantive validity [51].

##### Depressive Symptoms

Depression is assessed with the Patient Health Questionnaire–9 (PHQ-9) [52]. It consists of 9 items on a 4-point Likert scale, with a total score ranging from 0 to 27. Higher scores indicate higher levels of depression. The scores of 0-4, 5-9, 10-14, 15-19, and 20-27 are indicative of no depression, mild depression, moderate depression, moderately severe depression, and severe depression, respectively [52]. The PHQ-9 was found to have high sensitivity (0.71-0.84), specificity (0.90-0.97), internal consistency (Cronbach α=.86-.89), test-retest reliability (*r*=0.84), and validity [53,54].

##### Perceived Stress

Stress levels are assessed using the Perceived Stress Scale–10 (PSS-10) [55,56]. This self-report scale consists of 10 items on a 5-point Likert scale with a total score ranging from 0 to 40, where a higher score indicates higher perceived stress. The PSS-10 was found to have high validity, internal consistency (Cronbach α=.74-.91), and test-retest reliability (*r*=0.74-0.88) [56,57].

##### Quality of Life

Quality of life was measured using the Mental Health Quality of Life questionnaire (MHQoL) [58]. It contains 7 items on a 4-point Likert scale that cover the dimensions of self-image, independence, mood, daily activity, physical health, relationships, and future. The total score ranges from 0 to 21, where a higher score indicates a better quality of life. The MHQoL was found to have good validity, test-retest reliability (*r*=0.85), and internal consistency (Cronbach α=.85) [58].

##### Digital Coach Evaluation

The Working Alliance Inventory for guided Internet interventions (WAI-I) [59] is used to evaluate participants’ satisfaction with the e-coach. The WAI-I consists of 12 items on a 5-point Likert scale with a total score ranging from 12 to 60, where higher scores indicate higher satisfaction. The psychometric characteristics of the WAI-I yielded adequate results [59].

#### Additional Measures

The following sociodemographic information is collected to examine sample characteristics: age, gender, marital status, nationality, attending university, faculty, education level (bachelor, master’s, or PhD), and whether psychotherapy and medication are received.

#### Assessments

An overview of the assessments can be seen in Table 1.

**Table 1:** Measures and assessment points.

Measures	Assessment points		
	T0^a^	T1^b^	T2^c^
Sociodemographic	✓		
Client Satisfaction Questionnaire (CSQ-8)		✓	
System Usability Scale (SUS-10)		✓	
Irrational Procrastination Scale (IPS)	✓	✓	✓
Perceived Stress Scale (PSS-10)	✓	✓	✓
Patient Health Questionnaire (PHQ-9)	✓	✓	✓
The Mental Health Quality of Life Questionnaire (MHQoL)	✓	✓	✓

^a^Pretest.

^b^Posttest (4 weeks).

^c^Follow-up (6 months).

### Sample Size

There is no clear method to calculate the sample size of an open feasibility study. Studies have suggested sample sizes ranging from at least 12 participants [60,61] to 35 or more participants [62]. In this study, no power calculation was conducted to determine the sample size, as the main focus is on the feasibility and acceptability of the intervention. Based on these rules of thumb and similar previous studies [63], we anticipate that a minimum of 50 participants will be sufficient to examine our main objectives. However, as the intervention will remain accessible to students for several years as part of the service offering of the Caring Universities project, we anticipate more than 50 participants will make use of the intervention. We will analyze the data of all participants who have used the intervention at the time of data analysis to be able to draw stronger conclusions.

### Statistical Analysis

Participants’ satisfaction with the intervention, usability, and adherence will be examined by calculating descriptive statistics.

### Satisfaction

Each of the 8 items on the CSQ-8 has 4 answering options, of which 2 indicate dissatisfaction (scored 1 or 2) and the other 2 indicate satisfaction (scored 3 or 4). Our goal is for participants to be satisfied or very satisfied with the intervention, meaning a score of 3 or 4 per item. This corresponds with an average total score between 24 and 32. We will consider the intervention a success if average CSQ-8 scores reach a minimum of 24.

#### Usability

Bangor et al [64] have found that SUS-10 scores above 70 are passable, with scores above 90 indicating superior products. We will consider the intervention a success if average SUS-10 scores reach a minimum of 70.

#### Adherence

A recent meta-analysis looking at internet interventions for mental health among university students found dropout rates between 11.84% and 50.33% [65]. Given the nature of procrastination, dropout is expected to be on the higher side. We will consider the intervention a success if adherence reaches 50%.

#### Secondary Outcomes

We will conduct 2-tailed paired *t* tests using a significance level of α=.05 to assess changes in the scores of IPS, PHQ-9, PSS-10, and MHQoL. Average WAI-I scores will be calculated to assess satisfaction with the digital coach.

### Ethical Considerations

This study protocol was approved by the Scientific and Ethical Review Board of all universities that participate in Caring Universities on May 15, 2020 (Vrije University, Leiden University, Maastricht University, Utrecht University, Erasmus University, University of Amsterdam, and Inholland University of Applied Sciences; reference number 2020.088). Participants in the study provide informed consent to the collection, storage, and viewing of their personal data. If the researchers want to use the data later for further research and scientific education, or if interim interventions require new data to be processed or used in other ways than for which they were originally collected, this will be communicated to the participants, and permission will be asked again. Research data are collected by the platform, which complies with GDPR guidelines. These are then coded and shared with Vrije Universiteit Amsterdam, which will be responsible for the data processing. Identifying information, such as name and contact information, is stored separately from all other data that are collected. Each participant is assigned an ID code to which the collected data are linked. This ID code is stored separately and is only accessible to the researcher if it is necessary for linking data for contact or permission. The coded data will be analyzed within the study.

## Results

This study was funded in 2019, and recruitment began in January 2021. As of May 2023, a total of 985 participants were enrolled, of which 372 had completed the posttest and 192 had completed the follow-up. The expected date of analysis and publication of the results is 2024.

## Discussion

### Overview

This study aims to examine the feasibility and acceptability of a new, guided web-based intervention for procrastination in college students. The main outcomes are client satisfaction, usability, and adherence to the intervention. In addition, we will explore any changes in the secondary outcomes of procrastination, depressive symptoms, stress levels, and quality of life from pretest to posttest. We expect the intervention to be perceived as feasible and acceptable by college students. An improvement in the secondary measures upon receiving the treatment is also expected, though not all may reach significance.

### Strengths and Limitations

eHealth interventions targeting procrastination for students show promising results [26-30]. Guidance from professionals seems to increase the effectiveness of eHealth treatment when compared with unguided treatment [24,34]. However, to our knowledge, research on guidance by clinical psychology students in eHealth interventions is scarce in the literature. Therefore, it is our belief that this study will be informative in the development and implementation of eHealth interventions targeting procrastination among students in the Netherlands.

There are some limitations to be taken into consideration. First, the recruitment method must be mentioned. The main recruitment channel will be through a yearly web-based mental health survey of the WMH-ICS, which is a voluntary survey that is sent out to all students of participating universities. This may result in a selection bias, as not all students are equally likely to fill out the survey and subsequently participate in a voluntary web-based intervention. The sample in this study may therefore not be entirely representative of the Dutch student population. However, it is important to note that participants are not offered course credits or any other form of compensation. As a result, this study will provide an opportunity to examine the real-life demand and use of the intervention by students in 7 different universities across the Netherlands. While the sample may not be representative of the Dutch student population, it is still likely a good representation of students that seek help and are open to eHealth solutions.

Second, this study is a feasibility and acceptability study without a control group. As a result, any findings on the secondary measures of procrastination, depression, stress, and quality of life cannot be directly attributed to the intervention. Further research and randomized controlled trials will be necessary to draw conclusions on intervention effectiveness.

Lastly, nonadherence is a known problem in eHealth, which may be exacerbated by the nature of procrastination [28,31-33]. To increase adherence to the intervention, participants will receive guidance from trained clinical psychology students. This is expected to increase engagement, motivation, and adherence to the intervention [35-37].

There are also several strengths of this study. First is the design of the intervention, which is done in collaboration with students. The visual style of the intervention as well as its contents are discussed with students during the development. Student feedback is implemented to ensure that the intervention meets the needs of the student population. To further promote engagement and adherence, we design the intervention based on principles of optimized user interface [40] and work closely with a user experience designer. This resulted in a highly user-friendly platform, which is expected to promote students’ satisfaction and engagement with the intervention. Lastly, we include automated reminders as well as personal ones by the e-coaches to increase adherence [66].

The comprehensiveness of assessment is a second strength. While this study examines the acceptability and feasibility of a new eHealth intervention targeting procrastination, extensive secondary outcomes are also measured. Procrastination is linked to many mental health issues, such as depression, social anxiety, stress, and low self-esteem [8-11]. In this study, we will explore any changes in procrastination, depression, stress, and quality of life, as well as assess the main outcomes of adherence, client satisfaction, and usability. In doing so, we will gain a more comprehensive understanding of the intervention. It also allows us to explore potential moderators and mediators of intervention adherence and effectiveness.

Finally, we believe the use of trained clinical psychology students is a strength of this study. Guidance by nonclinicians such as peers, research assistants, and laypersons has been found to be equally effective as that by clinicians [38]. To our knowledge, no studies have been conducted on guidance by trained clinical psychology students. The use of student coaches is highly cost-effective, as clinical psychology students are often required to gain work experience through extracurricular internships during their studies. Therefore, student coaching would be a more scalable form of guidance. If student guidance in eHealth interventions was found to be feasible and acceptable, it could greatly reduce the load on the health care system and offer more timely access to mental health support to those in need.

### Conclusions

This study aims to examine the feasibility and acceptability of an eHealth intervention for procrastination specifically designed for college students and under the guidance of clinical psychology students. The results are expected to contribute to the body of literature regarding eHealth in several ways. First, the nature of procrastination might intensify the known problems of eHealth, such as nonadherence. This study examines whether students who procrastinate will adhere to and be satisfied with eHealth interventions targeting this problem. Second, this study explores whether an intervention targeting procrastination can also decrease depression and stress. Lastly, this pilot investigates whether trained psychology students can effectively guide their peers in web-based interventions. Given the shortage of licensed psychologists, exploring alternative sources of guidance is much needed in order to provide students with the mental health support they need.

## Abbreviations

CBT: cognitive behavioral therapy
CSQ-8: Client Satisfaction Scale–8
GDPR: General Data Protection Regulation
IPS: Irrational Procrastination Scale
MHQoL: Mental Health Quality of Life questionnaire
PHQ-9: Patient Health Questionnaire–9
PSS-10: Perceived Stress Scale–10
SUS-10: System Usability Scale–10
WAI-I: Working Alliance Inventory for guided Internet interventions
WHO: World Health Organization
WMH-ICS: World Mental Health International College Student Initiative

## Data Availability

The data sets generated during and/or analyzed during this study are available from the corresponding author on reasonable request.
